# Specificity in diversity: single origin of a widespread ciliate-bacteria symbiosis

**DOI:** 10.1098/rspb.2017.0764

**Published:** 2017-07-12

**Authors:** Brandon K. B. Seah, Thomas Schwaha, Jean-Marie Volland, Bruno Huettel, Nicole Dubilier, Harald R. Gruber-Vodicka

**Affiliations:** 1 Max Planck Institute for Marine Microbiology, Celsiusstraße 1, 28359 Bremen, Germany; 2 Department of Integrative Zoology, University of Vienna, Althanstraße 14, 1090 Vienna, Austria; 3 Department of Limnology and Bio-Oceanography, University of Vienna, Althanstraße 14, 1090 Vienna, Austria; 4 Max Planck Genome Centre Cologne, Max Planck Institute for Plant Breeding Research, Carl-von-Linné-Weg 10, 50829 Cologne, Germany; 5 MARUM, Center for Marine Environmental Sciences, University of Bremen, 28359 Bremen, Germany

**Keywords:** symbiosis, thiotrophy, ciliate, Gammaproteobacteria

## Abstract

Symbioses between eukaryotes and sulfur-oxidizing (thiotrophic) bacteria have convergently evolved multiple times. Although well described in at least eight classes of metazoan animals, almost nothing is known about the evolution of thiotrophic symbioses in microbial eukaryotes (protists). In this study, we characterized the symbioses between mouthless marine ciliates of the genus *Kentrophoros*, and their thiotrophic bacteria, using comparative sequence analysis and fluorescence *in situ* hybridization. Ciliate small-subunit rRNA sequences were obtained from 17 morphospecies collected in the Mediterranean and Caribbean, and symbiont sequences from 13 of these morphospecies. We discovered a new *Kentrophoros* morphotype where the symbiont-bearing surface is folded into pouch-like compartments, illustrating the variability of the basic body plan. Phylogenetic analyses revealed that all investigated *Kentrophoros* belonged to a single clade, despite the remarkable morphological diversity of these hosts. The symbionts were also monophyletic and belonged to a new clade within the Gammaproteobacteria, with no known cultured representatives. Each host morphospecies had a distinct symbiont phylotype, and statistical analyses revealed significant support for host–symbiont codiversification. Given that these symbioses were collected from two widely separated oceans, our results indicate that symbiotic associations in unicellular hosts can be highly specific and stable over long periods of evolutionary time.

## 1. Introduction

Symbiotic associations between eukaryotes and sulfur-oxidizing (thiotrophic) bacteria have evolved several times in different groups of both hosts and symbionts [1,2]. Among metazoan animals, they have evolved independently in at least eight taxonomic classes. By contrast, much less is known about thiotrophic symbioses in protists (microbial eukaryotes), with only two groups described as hosts, namely euglenozoans [3] and ciliates [4]. The thiotrophic symbionts of animals and protists fall in several clades of bacteria: mostly Gammaproteobacteria [1], but also Epsilon- [5] and Alphaproteobacteria [6]. Many are interpreted as nutritional symbioses because the hosts have reduced digestive systems, and the symbionts can use energy from inorganic reduced sulfur to produce new biomass from CO_2_.

*Kentrophoros* (Ciliophora: Karyorelictea) is a genus of ciliates with two unusual characters: lack of a differentiated cytostome (oral apparatus, or ‘mouth’), and an obligate association with ectosymbiotic thiotrophic bacteria [7–9]. The cell body is flattened, with one side ciliated and the other densely covered by the bacteria. The symbionts of *Kentrophoros* are sulfur oxidizers (thiotrophs) [10] and are phagocytosed by the ciliates directly along the whole cell body [8,11,12]. Of the ciliates known to have thiotrophic symbionts—*Kentrophoros*, *Zoothamnium niveum* [4] and possibly *Pseudovorticella* sp. [13]—only the symbionts of *Z. niveum* have been phylogenetically identified. *Zoothamnium niveum* is a single representative in a predominantly non-symbiotic genus. By contrast, *Kentrophoros* is a genus comprising many species that all bear thiotrophic symbionts. These hosts are geographically widespread in marine sediment interstitial habitats (references in [9]) and can be locally abundant [14], and are thus valuable for comparing the biology and evolution of symbiotic associations within a speciose group of hosts.

The symbiotic bacteria have remained unidentified, although they were described a long time ago [15,16]. It is not known whether the *Kentrophoros* symbionts are all close relatives to each other or if they come from different clades, nor is it possible to infer from morphology and physiology alone if they are related to known groups of thiotrophic bacteria. They may represent one or more entirely new clade(s) of symbiotic thiotrophs. The identity of the symbionts also relates directly to the question of host–symbiont specificity. Some clades of thiotrophic symbionts have a very specific relationship to their hosts, even exhibiting a pattern of codiversification [6], while others are associated with two or more different host taxa or have close relatives that are non-symbiotic [17].

The remarkable morphological diversity of *Kentrophoros* has also called their own phylogenetic position into question. The described species differ widely in size and body shape, as well as the number and arrangement of nuclei. The genus might therefore be polyphyletic, i.e. mouthlessness and symbiotic lifestyle may have evolved more than once among the karyorelict ciliates [9]. Alternatively, *Kentrophoros* may simply be more variable than other ciliate genera. Molecular phylogenetics can help to resolve such taxonomic problems when morphology is difficult to interpret, but only two 18S rRNA sequences have been published [18,19]. The true extent of *Kentrophoros* species diversity is also unclear because karyorelictean ciliates are notoriously difficult to handle [9,20], and most descriptions have been exclusively morphological.

In this study, we collected *Kentrophoros* from two geographical regions, the Mediterranean and Caribbean Seas, to identify the symbionts and test if the symbiosis had a single origin. More specifically, we ask: (i) is *Kentrophoros* a monophyletic group within the karyorelict ciliates? (ii) Do the symbiotic bacteria also form a monophyletic group, and are they related to known groups of symbiotic bacteria? (iii) How specific and stable are these associations, and have hosts and symbionts co-diversified? (iv) How does the morphological diversity of *Kentrophoros* relate to phylogeny? To address these questions, we used methods from molecular ecology, phylogenetics and comparative morphology.

## 2. Material and methods

### (a) Sampling site and collection

Mediterranean samples of *Kentrophoros* were collected in 2013 and 2014 from three localities off the island of Elba, Italy. At the bays of Cavoli (42.734192° N, 10.185868° E, 12.8 m depth) and Sant’ Andrea (42.808561° N, 10.142275° E, 7.3 m depth), ciliates were extracted by decanting sandy sediment collected by scuba divers. At Golfo di Barbatoia off Fetovaia, Elba (42.7313° N, 10.1534° E, 1.5 m depth), sediment was collected in Plexiglas cores by snorkelling and extracted by the Uhlig method [21]. Caribbean samples were collected in 2015 off the southern end of Twin Cayes, Belize (16.82356° N, 88.106150° W, 1.5 m depth), by both decantation and Uhlig extraction.

### (b) DNA extraction

Samples for DNA extraction were either fixed in RNAlater (Sigma-Aldrich) (stored at 4°C) or 70% ethanol (stored at −20°C) or directly digested in buffer ATL and proteinase K of the DNeasy Blood and Tissue kit (Qiagen). DNA was extracted from single *Kentrophoros* cells with the DNeasy Blood & Tissue Mini Kit following the manufacturer's protocol, and eluted in 50 µl elution buffer.

### (c) Sequencing of *Kentrophoros* 18S rRNA gene

The 18S rRNA gene was amplified by polymerase chain reaction (PCR) with general eukaryote primers EukA (AACCTGGTTGATCCTGCCAGT) and EukB (TGATCCTTCTGCAGGTTCACCTAC) [22] using Phusion high-fidelity DNA polymerase (Thermo), 50 µl reaction volume with 1 µl template, and touchdown thermocycle: 98°C/2 min—10 cycles of (98°C/10 s—70°C (reduced by 1°C per cycle)/30 s—72°C/1 min)—30 cycles of (98°C/10 s—60°C/30 s/72°C/1 min)—72°C/10 min—held at 12°C. PCR product bands were cut from the gel after electrophoresis, purified with the Qiaquick gel extraction kit (Qiagen) and sequenced with BigDye Terminator v 3.1 Cycle Sequencing Kit (Life Technologies) on a 3130 × 1 Genetic Analyzer (Applied Biosystems), using EukA, EukB and 18SF492karyo (AGGACCCACTGGAGGG, modified from [23]) as sequencing primers. Sequence chromatograms were inspected and assembled in Sequencher 4.6 (Gene Codes), retaining sequences that had more than 95% of bases with a Phred score greater than 20. PCR products that could not be directly sequenced were cloned before sequencing, using the TOPO TA kit (Invitrogen) with pCR-4-TOPO vector and One-Shot TOP10 *Escherichia coli* chemically competent cells, after adding A-overhangs with Taq polymerase (5 Prime) and dATP. Vector primers M13F and M13R were used as sequencing primers for clones.

### (d) Sequencing of symbiont 16S rRNA gene

Metagenomic sequencing libraries were prepared from *Kentrophoros* morphospecies H, SD, LPFa, LFY, TUN and UNK with the Ovation Ultralow Library System V2 (NuGEN) following the manufacturer's protocol. Libraries were sequenced on the Illumina HiSeq 2500 platform as 100 bp paired-end reads, with approximately 10 million reads per library. The 16S rRNA sequences were reconstructed with the phyloFlash pipeline (https://github.com/HRGV/phyloFlash): reads with greater than 70% identity to reference 16S rRNA sequences were extracted by BBMap (https://sourceforge.net/projects/bbmap/), and assembled with EMIRGE [24] or SPAdes [25]. The 16S rRNA sequences with the highest read coverage per library were considered candidate symbiont sequences. The candidate symbiont 16S rRNA genes from the above six host morphospecies were aligned and used to design two sets of PCR primers to amplify symbiont 16S rRNA sequences from the remaining host morphospecies: chr4Amix (CGAACGGTAACGGGGGGA, CGAACGGTAACGGGGGAA, CGAACGGTAACGGAGGGA) and chr4Cmix (CCGAGGATGTCAAAAGCAGG, CCAAGGATGTCAAAAGCAGG). PCR was performed with primer pairs chr4Amix/1175R (CGTCATCCMCACCTTCCTC, [26]) or b341 (CCTACGGGAGGCAGCAG, [27])/chr4Cmix using Phusion polymerase, 20 µl volume with 2 µl template, and a touchdown thermocycle: 98°C/2 min—15 cycles of (98°C/10 s—70°C (reduced by 1°C per cycle)/30 s—72°C/1 min)—25 cycles of (98°C/10 s—55°C/30 s/72°C/1 min)—72°C/10 min—held at 12°C. PCR products were purified and sequenced as described above.

### (e) Molecular phylogenetics

For both ciliate 18S rRNA and bacterial 16S rRNA sequences, a similar protocol was used. Sequences were dereplicated at 99% identity with Usearch [28] (cluster_fast, length-sorted). Outgroup sequences were downloaded from SILVA SSU Ref NR 123 [29] or NCBI GenBank (accession numbers in electronic supplementary material) and aligned with sequences from this study using the L-INS-i method in MAFFT 7.130b [30]. The best-fitting evolutionary model, GTR+*Γ* in both cases, was found with jModelTest2 [31] from 44 alternatives. Phylogenies were estimated with four discrete rate categories. Maximum-likelihood estimation was performed with RAxML v. 8.1.3 [32] using the rapid hill-climbing algorithm, 10 randomized starts and Shimodaira–Hasegawa-like (SH-like) support values from the approximate likelihood ratio test (aLRT) [33]. Bayesian inference was performed with MrBayes v. 3.2.5 [34] using two independent runs of four Monte Carlo Markov Chains (three heated, one cold) for 5 × 10^6^ (18S rRNA) or 10 × 10^6^ (16S rRNA) generations, with 25% relative burn-in. For the 16S rRNA tree, the maximum-likelihood tree was used as a starting tree to improve convergence. Potential scale reduction factor values between 0.99 and 1.01 indicated convergence. For the 16S rRNA tree, an initial run gave inconsistent results between maximum likelihood (ML) and Bayesian trees, and poor convergence in the Bayesian analysis. Potential rogue taxa were identified with RogueNaRok v. 1.0 [35] on 500 bootstrap replicates estimated on the original alignment with RAxML. Rogue taxa that were not candidate symbiont sequences were removed, and the phylogenetic analyses were repeated with the previous parameters.

### (f) Host–symbiont codiversification analysis

Bayesian trees of host and symbiont small subunit (SSU) rRNA were used for codiversification analysis. Host morphospecies for which the corresponding symbiont sequences were unavailable were removed from the tree without changing other branches, as required by the software tools used. Event-based analysis with Jane v. 4 [36] used the default cost scheme and ran the genetic algorithm for 20 generations with population size 100. Random sampling for significance testing used random tip mapping and sample size 100. Distance-based analysis with PACo [37] used distance matrices calculated from the edited host and symbiont trees. A total of 10^5^ iterations of random permutation were used for significance testing.

### (g) Fluorescence *in situ* hybridization

Formaldehyde-fixed specimens of *Kentrophoros* morphospecies H were dehydrated in ethanol (70, 80, 95, 95, 100, 100, 100%, more than 30 min per step), transferred twice through Roti-Histol (Carl Roth) (more than 1 h per step), 1 : 1 mixture of Roti-Histol and Paraplast paraffin (60°C, 1 h) and six times through paraffin (60°C, more than 1 h per step). The paraffin block was solidified at room temperature for one week. Sections were cut on a Leica RM2165 microtome at approximately 5 µm thickness, floated onto glass slides (Superfrost Plus) and baked (56°C, 2 h). Sections were dewaxed (3 × Roti-Histol, more than 30 min, room temperature) and rehydrated (ethanol 96, 80, 70%).

Specific probes chr4Ca (CCGAGGATGTCAAAAGCAGG) and chr4Ba (GTAGGCTCATCCAACAGC) were designed in ARB [38]; chr4Ca targets five candidate symbiont phylotypes with zero mismatches, and three with one mismatch (out of nine phylotypes with coverage of the probe target region), whereas chr4Ba targets only the candidate symbiont phylotype from *K*. sp. H with zero mismatches. Matches to published sequences were checked with TestProbe versus the Silva SSU Ref NR 123 database [29]. All zero-mismatch hits to chr4Ca and chr4Ba were uncultivated environmental sequences, numbering 11 and 5, respectively. No database sequence had matches to both chr4Ca and chr4Ba. For probe chr4Ca, unlabelled ‘helper’ oligonucleotides (TAAGGTTCTTCGCGTTGCAT, CGTGTGTAGCCCTGCCCATA, CGTGTGTAGCCCTGCTCATA) were designed, which bind to adjacent regions in the rRNA and improve the primary probe signal [39]. Different formamide concentrations were tested in the hybridization buffer with and without helpers, on paraffin-embedded sections of *K.* sp. H. Final formamide concentrations used were 20% for chr4Ca and 40% for chr4Ba. Probe specificity was tested against *Beggiatoa* sp. 35Flor for chr4Ca (three mismatches) and with cloneFISH [40] for chr4Ba (one mismatch), with NON338 as negative control.

Catalysed reporter-deposition fluorescence *in situ* hybridization (CARD-FISH) was performed as described by [41] with fluorophore Alexa 488 (Life Technologies) except that hybridization and washing were performed at 46 and 48°C, respectively, and an additional lysozyme permeabilization step was included [42].

**Figure 1.**
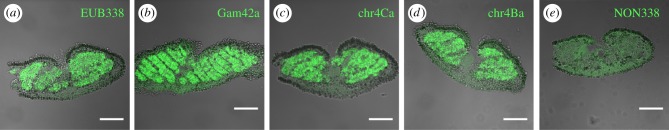
FISH of *Kentrophoros* sp. H cross-sections with oligonucleotide probes targeting bacterial rRNA. Emission in 508–534 nm channel from fluorophore Alexa 488 (excitation 488 nm) overlaid on transmitted light image. Probes match sequence signatures specific to successively more exclusive groups: (*a*) EUB338—most Bacteria (positive control), (*b*) Gam42a—most Gammaproteobacteria, (*c*) chr4Ca—symbionts of several *Kentrophoros* species, (*d*) chr4Ba—symbiont of *Kentrophoros* sp. H only, (*e*) NON338—reverse complement of the general bacteria probe (negative control). Scale bars, 25 µm. (Online version in colour.)

*Kentrophoros* sp. H sections from two individuals were separately hybridized with four different probe sets of increasing taxonomic specificity: EUB338I-III targeting most Bacteria [43,44], Gam42a (with unlabelled Beta42a competitor) targeting most Gammaproteobacteria [45], chr4Ca (with unlabelled helper probes) targeting most *Kentrophoros* candidate symbiont sequences and chr4Ba, targeting only the candidate symbiont of *K.* sp. H. Slides were viewed under epifluorescence with a Nikon Eclipse 50i microscope, Intensilight C-HGFI light source (Nikon) and filter F46-018 (AHF Analysentechnik). Imaging for figure 1 was performed on a Zeiss LSM 780 confocal laser-scanning microscope with 63× Plan-Apochromat oil-immersion objective, excitation 488 nm, emission 508–534 nm.

### (h) Histology and three-dimensional reconstruction

Samples for semithin sections were fixed in 1% OsO4 buffered with 0.1 M sodium cacodylate, 1100 mOsm l^−1^, pH 7.4 (Electron Microscopy Sciences) for 2 h, washed three times in the same buffer, post-fixed with a mixture of 2.5% glutaraldehyde and 2% formaldehyde in the same buffer overnight (more than 12 h), washed three times with distilled water, dehydrated in ethanol (30, 50, 70%) and stored in 70% ethanol until use. All steps were carried out on ice or at 4°C. Fixed specimens were dehydrated in an ethanol series and embedded in EMBed 812 resin (Electron Microscopy Sciences) using acetone as intermediate solvent. The resin was mixed in the ‘hard’ formulation and cured at 60°C for 24 h. Blocks were serially sectioned at 1 µm thickness on a Leica UC6 ultramicrotome (Leica Microsystems, Wetzlar, Germany). Sections were stained with toluidine blue and photographed with an Axiocam colour camera mounted on a Zeiss Axio Image A1 microscope (Zeiss, Oberkochen, Germany). Semithin sections for three-dimensional reconstruction were sealed in resin and photographed with a DP73 camera on an Olympus BX53 compound microscope (Olympus, Tokyo, Japan). Section images were converted to greyscale with Adobe Photoshop CS5 (Adobe, San Jose, CA, USA). Each image stack was imported into the three-dimensional reconstruction software Amira 6.0 (FEI, Hillsboro, OR, USA), and aligned with the AlignSlices tool. Aligned stacks were semi-automatically segmented with threshold segmentation, followed by manual corrections. Specimens were visualized by volume rendering of the original image stack or surface rendering of the segmentation. Volumes of the segmented areas (entire body, symbiont region and nuclei) were measured with the ‘measurement’ option in Amira.

## 3. Results

### (a) *Kentrophoros* is a monophyletic genus despite its morphological diversity

**Figure 2.**
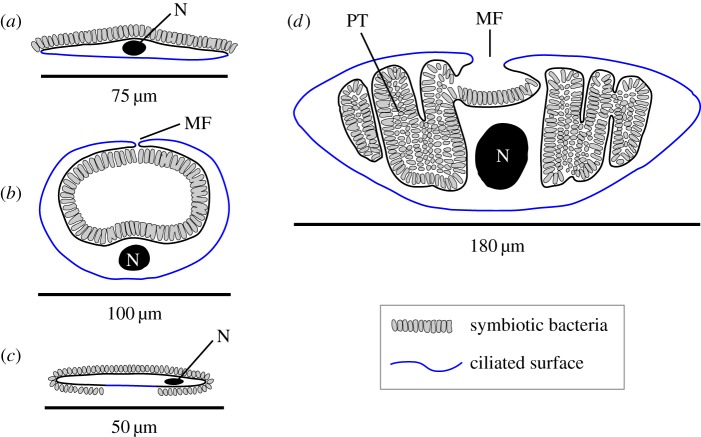
Schematic diagram of transverse sections illustrating different body involution types in *Kentrophoros:* (*a*) ‘open’, non-involuted (e.g. *K. fasciolatus*), (*b*) ‘tubular’ involution (e.g. *K. fistulosus*), (*c*) ‘*canalis*-type’ with symbionts on part of ventral surface (*K. canalis*), (*d*) ‘pseudotrophosomal’ with pocketing of symbiont-bearing surface (*K*. sp. H). N, nucleus; MF, medial furrow; PT, pseudotrophosome. (Online version in colour.)

Specimens of *Kentrophoros* were identified in the field by their dense ectosymbiont coat, and sorted into 17 putative morphospecies by host characters observable in live organisms, especially overall body shape, size, and whether the cell body was rolled up (involuted) (electronic supplementary material, table S1; figure 2). Each morphospecies was given a placeholder identifier (electronic supplementary material, table S1).

Five *Kentrophoros* morphospecies appeared to have more than one 18S rRNA sequence per genome. Their PCR products consistently yielded overlapping chromatograms when directly sequenced, suggesting that they were mixtures of different sequences, although PCR was performed on single-cell samples. For each of these morphospecies, PCR products from two specimens were separately cloned for sequencing. Cloned sequences from the same individuals not only had substitutions but also insertion–deletion polymorphisms, which is consistent with the difficulty in sequencing the initial PCR product directly.

**Figure 3.**
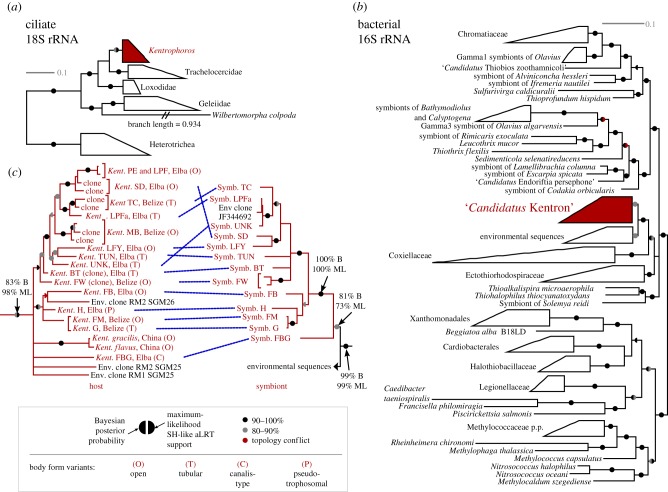
Small-subunit rRNA phylogenies of host (*a*) and symbiont (*b*) species, with detail of representative sequences (see §2) from the *Kentrophoros* and *Ca.* Kentron clades (*c*). Trees from Bayesian inference are displayed, with support values from both Bayesian and maximum-likelihood analyses (see Key) on branches. In (*c*), blue lines connect host–symbiont pairs, body involution type of host morphospecies is indicated by letters in parentheses (see key), and other uppercase letters are identifiers for *Kentrophoros* morphospecies (see table S1). Full trees available online at TreeBASE (S19762). Scale bars: substitutions per site. (Online version in colour.)

*Kentrophoros* sequences from this study fell into a single clade with the two published *Kentrophoros* sequences [18] and three environmental clone sequences from deep-sea cold seep sediments in Sagami Bay, Japan, that were previously of uncertain affiliation [46] (figure 3). The clade was well-supported in the maximum-likelihood analysis (98% SH-like aLRT) but only moderately so in the Bayesian analysis (83% posterior probability). The Trachelocercidae were recovered as the sister group to *Kentrophoros*, with weak to moderate support (74% Bayesian, 60% maximum likelihood). Within *Kentrophoros*, however, many internal branches were short and some species relationships were poorly resolved, although there were some well-supported species clusters. Two morphospecies from the same locality in Belize, *Kentrophoros* spp. FM and G, differed by only 3 bp (in 1360 bp alignment), but these substitutions were consistently associated with their morphospecies identification (four individuals each sequenced).

The 18S rRNA sequences corresponded well to their morphospecies identification, for both direct and cloned sequences, with two exceptions. (i) Morphospecies *Kentrophoros* sp. SD required cloning, and the resulting clones were represented by two representative sequences when clustered at 99% identity. However, the representatives did not form a monophyletic cluster. (ii) Sequences from morphospecies *K.* spp. LPF, PF and PFC clustered together with high identity (greater than 98%, resulting in two representative sequences after clustering at 99% identity), but the clustering did not correspond to their assigned morphospecies. This suggests that the 18S rRNA gene had insufficient resolution, or that the morphological sorting was imperfect.

### (b) Symbionts of *Kentrophoros* are a new lineage of thiotrophic symbionts

The 16S rRNA sequences from the symbionts, as confirmed later by FISH (see below), were obtained by metagenomic sequencing from six host morphospecies, and by PCR amplification with specific primers (approx. 600 bp region) from a further seven. PCR was not successful for four morphospecies (electronic supplementary material, table S1). The minimum sequence identity among symbiont sequences was 93%. The top-scoring hits to the SILVA SSU Ref NR 123 database [29] were all uncultivated environmental sequences. The best hits with more than 90% identity were included in our analysis, along with cultivated strains representing each taxonomic order in basal Gammaproteobacteria, and known thiotrophic symbionts.

The symbiont sequences fell within the basal Gammaproteobacteria, forming a well-supported clade (99% Bayesian, 99% maximum likelihood) with environmental sequences. Within this clade, the symbionts alone formed a moderately well-supported clade (81% B, 73% ML), and if the most basal symbiont (from *K.* sp. FBG) was excluded, the remaining group was highly supported (100% B, 100% ML). An environmental sequence from marine sediment (JF344692) fell among the symbionts, while the other environmental sequences, which were from marine sediment or coral-associated, formed a separate cluster (84% B, 83% ML). The next closest relatives were the Coxiellaceae (92% B, 83% ML), followed by the Ectothiorhodospiraceae (90% B, 92% ML). The symbionts diverged from other known thiotrophic symbiotic bacteria, including *Ca.* Thiobios zoothamnicoli, the only other well-characterized thiotroph symbiont from a ciliate host.

Symbiont sequences from the same host morphospecies always clustered together or collapsed to the same representative sequence (at 99% identity) (figure 3). Host and symbiont phylogenies significantly supported codiversification under two different types of analysis. Event-based analysis, which considers only tree topology, predicted 10 cospeciation and two host-shift events, with an optimal total cost of 8, significantly less (*p* = 0.0) than the cost of randomized trees (mean 27.3, standard deviation 4.9). Distance-based Procrustean analysis, which uses branch length information, yielded a goodness-of-fit metric *m*^2^ = 0.0157, significantly better (*p* = 0.0) than the metrics for randomized associations (mean 0.062, standard deviation 0.0053). Nonetheless, host and symbiont phylogenies were not strictly congruent. For example, symbiont sequences from two host morphospecies, *K.* spp. UNK and LPFa, were more than 99% identical, even though their host 18S rRNA sequences were not closely related (figure 3).

FISH confirmed that the 16S rRNA sequence recovered from *Kentrophoros* sp. H came from the bacterial cells covering its surface. The following oligonucleotide probes were used: Gam42a targeting the Gammaproteobacteria in general, chr4Ca targeting most symbiont sequences and chr4Ba targeting only the symbiont sequence from morphospecies *K.* sp. H. All probes gave an unambiguous signal corresponding morphologically to the symbiotic bacteria, comparable in intensity to the positive control probe EUB338I-III, which binds to all bacteria (figure 1).

We propose the name ‘*Candidatus* Kentron eta’ for the bacterial ectosymbiont of *Kentrophoros* morphospecies H, with *Ca.* Kentron comprising the thiotrophic symbionts of *Kentrophoros* ciliates in general. The assignment is based on the symbiont 16S rRNA sequence (accession LT621987), and hybridization with oligonucleotide probes chr4Ca and chr4Ba. The generic name (nom. neut. sing.) means ‘spine’ in Greek and is the first half of the host genus name, while the species name (irreg. neut. indecl.) is from the Greek progenitor of the Latin letter H. Morphologically, all known *Ca.* Kentron are rod-shaped bacteria, containing refractile globules that are presumably elemental sulfur, and exhibiting cell division along the longitudinal, rather than transverse, axis [8,9,12,16].

### (c) Diversity of the symbiotic body plan

To document the morphological diversity of *Kentrophoros* hosts, we focused on cell body involution, which can be directly observed in live specimens in the field. At both the Mediterranean and Caribbean sites, we found three types of involution that have been described previously: (i) ‘open’—cell body flattened and ribbon-like, ventral side ciliated and the dorsal side bearing ectosymbionts, e.g. the type species *Kentrophoros fasciolatus* [7]; (ii) ‘tubular’—involuted into a tube, with the ectosymbiont-bearing dorsal side inside the tube, e.g. *Kentrophoros fistulosus* [8,9]; (iii) ‘*canalis*-like’—ectosymbiont coat extends beyond dorsal side over to the ciliated ventral side, leaving only a stripe down the middle that is ectosymbiont-free, so far only known in *Kentrophoros canalis* [47].

**Figure 4.**
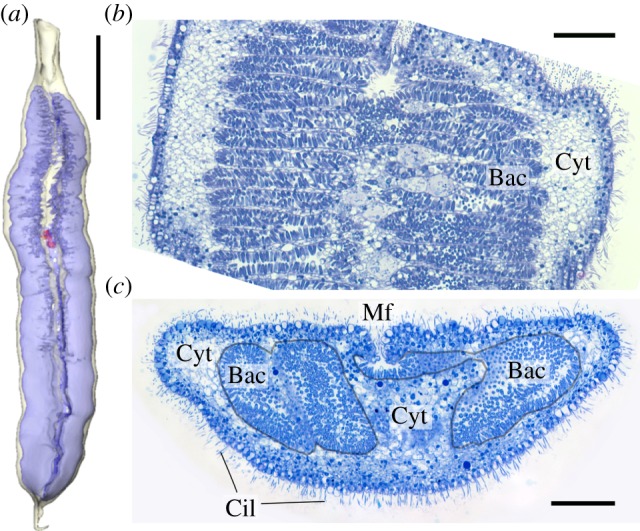
(*a*) Three-dimensional rendering of *Kentrophoros* sp. H reconstructed from serial sections. Highlighted volumes: off-white—cell outline; blue—symbiont-bearing pseudotrophosome; red—nuclei of the ciliate. Scale bar, 200 µm. (*b*) Longitudinal section of *K*. sp. H, stained with toluidine blue. Scale bar, 20 µm. (*c*) Transverse section of *K*. sp. H, with pseudotrophosome outlined in grey. Scale bar, 20 µm. Each image is from a different individual. Bac, bacteria; Cyt, ciliate cytoplasm; Mf, median furrow; Cil, cilia. (Online version in colour.)

A new type of cell body involution was observed in morphospecies *Kentrophoros* sp. H from Elba. The entire body was involuted except for the anterior and posterior extremities (‘head’ and ‘tail’), with the symbiont-bearing surface on the inside. Moreover, the bacteria appeared to be packed into a regular series of pouches, projecting laterally from the median axis. Serial sections of two entire individuals showed that the pouches were formed by folds and undulations of the symbiont-bearing surface, but that the surface was contiguous and did not form closed-off chambers (figure 4). In analogy to the endosymbiont-bearing trophosome body region in animals such as the tubeworm *Riftia pachyptila*, we call the symbiont-filled region of morphospecies *K.* sp. H the ‘pseudotrophosome’, because the symbionts appear enclosed but are still topologically outside the host cell body. The pseudotrophosome occupies 50% of the total volume of the symbiotic organism, as estimated from three-dimensional reconstruction of a complete, serially sectioned individual (figure 4).

For some morphospecies, there was adequate material to characterize the number and arrangement of nuclei by staining with the DNA-specific dye DAPI (electronic supplementary material, table S1). These were also diverse: the nuclei were arranged in a single loose row, or in clusters. Some had a single cluster, while others had multiple clusters arranged in a row, and the numbers of micro- and macronuclei per cluster could also differ. The nuclear configurations observed in our samples corresponded to many of those already described in published species (summarized in [48]). Based on the nuclei and body shape, a tentative identification was made for two of the morphospecies we collected on Elba: *K.* sp. FBG with *K. canalis*, and *K.* sp. PFC with *K. uninucleatus*.

No clear phylogenetic pattern was observed when either cell body involution types or nuclear characters were mapped onto the 18S rRNA phylogeny (figure 3; electronic supplementary material, table S1). For example, the clade containing *K.* spp. FB, H, FM and G has members with open, tubular and pseudotrophosomal body shapes; one has only three nuclei per cell (*K.* sp. H), while the others have more than 10.

## 4. Discussion

We have presented evidence for a single origin of the *Kentrophoros* symbiosis among both the hosts and symbionts. *Kentrophoros* specimens belonging to different morphospecies and collected from two well-separated localities, the Mediterranean and the Caribbean, fell in the same 18S rRNA clade. Their associated symbionts formed a new distinct clade, which we have named *Ca*. Kentron. Moreover, the ciliates are more morphologically diverse than previously anticipated, with a new variant on the *Kentrophoros* body plan discovered during this study. This is only the second group of ciliates and third group of protists for which thiotrophic symbionts have been phylogenetically identified. It is now clear that thiotrophic symbioses have evolved independently in these three protist groups, as well as in their symbionts, which belong to phylogenetically distinct lineages in Gamma- and Epsilonproteobacteria [3,4]. Our results highlight the relevance of microbial eukaryotes as hosts for such symbioses, and we predict that many more thiotrophic symbioses remain to be discovered in protists.

### (a) Phylogenetic position of the symbiotic bacteria

The symbionts of *Kentrophoros* belong to the basal Gammaproteobacteria, which include ‘classical’ free-living thiotrophs such as *Beggiatoa*, and also thiotrophic symbionts of eukaryotes. Both thiotrophy and symbiosis have repeatedly evolved in basal Gammaproteobacteria [1] and many clades of thiotrophic symbionts are either affiliated with more than one clade of host organisms, or have free-living members [17,49,50]. The *Ca*. Kentron clade contains all known *Kentrophoros* symbionts but only one environmental sequence (JF344692) from anaerobic marine sediment, which is a habitat where *Kentrophoros* can be found, so it is likely that *Ca*. Kentron comprises only *Kentrophoros* symbionts.

Our phylogenetic analyses showed that *Ca*. Kentron represents an independent origin of thiotrophic symbiosis within the Gammaproteobacteria. The sister group to *Ca*. Kentron is a cluster of environmental sequences from sediment, sponges and corals. As these sequences come from environmental clone libraries with no information on how they were collected, we cannot determine whether they originated from *Kentrophoros*, symbionts of other hosts or free-living bacteria. However, the next closest relatives are obligate intracellular parasites (the Coxiellaceae) and free-living sulfur-oxidizers (Ectothiorhodospiraceae), and not other symbiotic thiotrophs.

### (b) Host–symbiont codiversification

The phylogenies of host and symbiont showed statistically significant evidence of codivergence. *Kentrophoros* is assumed to reproduce asexually by fragmentation or fission, so the symbionts would generally be inherited vertically by daughter cells. Codiversification between ectosymbiotic bacteria and motile eukaryotic hosts may seem surprising, as ectosymbionts are constantly exposed to their surrounding environment. However, recent studies have shown that the thiotrophic ectosymbionts of marine nematode worms [51] and the ectosymbionts of termite gut flagellates [52] have also cospeciated with their hosts, which highlights how codiversification and mechanisms for symbiont recognition and maintenance, previously assumed to be characteristic for endosymbioses, also occur in ectosymbioses.

The phylogenies of *Kentrophoros* and their symbionts are not strictly congruent (figure 3). Indeed, our analyses indicated that *Ca*. Kentron switched between host species at least twice. Strict host–symbiont codiversification patterns would also be disrupted if hosts take up hitherto unrecognized free-living *Ca.* Kentron strains from their environment [53]. *Kentrophoros* have not been cultivated, so symbiont-free life cycle stages that would also disrupt vertical transmission, such as cysts, cannot be ruled out, although cysts are not known from the karyorelict ciliates [54].

Our study adds to a growing body of evidence that most thiotrophic symbionts, including intracellular ones, have mixed modes of transmission [53]. Nonetheless, in *Kentrophoros*, both the host and symbiont clades remain specific and exclusive to each other, a pattern that has only been observed among thiotrophic symbioses in the flatworm *Paracatenula* [6] and the vesicomyid clams [55]. In other cases, a single symbiont clade may be associated with more than one host clade [51], or vice versa [56]. The apparently stable association between *Kentrophoros* and *Ca*. Kentron indicates that there are clade-specific recognition mechanisms (otherwise thiotrophic symbionts from other lineages would associate with *Kentrophoros*), in addition to species/strain-specific recognition (otherwise host switches would occur more often within *Ca*. Kentron).

The phylogenies may be even more congruent if not for the presence of multiple 18S rRNA sequence types in single cells of some *Kentrophoros* morphospecies. The different gene copies may undergo duplication, divergence and loss within a single organismal lineage, independently of speciation. Most eukaryotes have multiple rRNA gene copies, often in identical tandem repeats, but many cases of divergent paralogues have also been reported, particularly among the alveolates, the group that includes the ciliates [57]. Intra-individual diversity of rRNA gene copies has previously been demonstrated with single ciliate cells [58], but this is the first time that this has been shown for the karyorelicteans. The 18S rRNA gene is routinely used for ciliate taxonomy, but the tree is not well resolved at the species level, which illustrates some limitations of single-gene phylogenies. Having additional markers, such as mitochondrial genes [59], may circumvent some of these problems, but for most ciliate species, especially the karyorelicteans, the 18S rRNA gene is the only molecular marker available [60], so this would come at the cost of reduced taxon sampling.

### (c) Diversity of the host ciliates

The monophyly of *Kentrophoros* falsifies the hypothesis [9] that the genus is polyphyletic. Its morphological diversity can instead be interpreted as variants upon a basic body plan, which we postulate to be exemplified by a flat ribbon-like cell body, and an approximate 2 : 1 ratio of macro- to micronuclei (the ratio in most karyorelicts [61]). The extensive folding of the symbiont-bearing surface in *K.* sp. H into a pseudotrophosome represents a new body plan variant. This increases the available surface area for ectosymbiont attachment, despite the ciliate's large size, maintaining a high bacteria : holobiont biovolume ratio of 50%, comparable to smaller species such as *K. fistulosus* (53%, measured from fig. 1 of [8]) and *K.* cf. *flavus* (50%, [10]). This is higher than the ratio in the gutless flatworm *Paracatenula* (33–50%, [6]), which is the highest known from metazoans with thiotrophic symbionts. Given that the ciliate cytoplasm also contains digestive vacuoles with engulfed symbionts, the bacteria are arguably the dominant partner in terms of biomass.

The morphological diversity of the *Kentrophoros* symbiosis contrasts with the thiotrophic symbiosis hosted by marine interstitial stilbonematine nematodes (family Desmodoridae) [62,63]. In *Kentrophoros*, the hosts are diverse in body form, while the bacteria are consistently rod-shaped, whereas for the nematodes, the symbionts are diverse (small cocci to long unicellular filaments [62,64,65]), while the hosts are always more or less cylindrical and do not vary widely in size, although they have specializations in other characters such as the cuticle and sexual organs [66]. In both the *Kentrophoros* and nematode symbiotic systems, several species can co-occur in the same localities. Although the functional significance of the different morphologies is unclear, co-occurrence of related species may indicate niche differentiation at small spatial scales within the interstitial environment.

We argue that the symbiosis between *Kentrophoros* and *Ca*. Kentron is an adaptive radiation: it has a single phylogenetic origin but is speciose, geographically widespread and morphologically diverse, although we have likely only sampled a small fraction of its diversity. As a ciliate, *Kentrophoros* provides a contrast to the well-known metazoan-hosted models for thiotrophic symbiosis, and gives us the opportunity to explore functional and evolutionary parallels among disparate organisms with such a lifestyle.

## Supplementary Material

Supplementary Table 1

## Supplementary Material

Supplementary Table 2

## Supplementary Material

Supplementary Table 3

## Data accessibility

Sequences: European Nucleotide Archive (*Kentrophoros* 18S rRNA—LT621756 to LT621967; symbiont 16S rRNA—LT621968 to LT622020). Alignments and trees: TreeBASE (study number S19762). Three-dimensional imaging: Dryad: http://dx.doi.org/10.5061/dryad.nc5dp [67].
